# Prevalence and clinical characteristics of molar-incisor hypomineralization in Syrian children: a cross-sectional study

**DOI:** 10.1038/s41598-023-35881-3

**Published:** 2023-05-26

**Authors:** Zuhair Al-Nerabieah, Muaaz AlKhouli, Mayssoon Dashash

**Affiliations:** Paediatric dentistry department, Faculty of Dentistry, Damascus University, Damascus, Syria

**Keywords:** Diseases, Health care, Medical research

## Abstract

This study was undertaken to determine the prevalence of molar incisor hypomineralization (MIH) in Syrian children and to provide information about clinical patterns and severity of MIH lesions. A sample of 1138 children aged 8–11 years was recruited for this cross-sectional study. The diagnosis of MIH was made using the criteria of the European Academy of Paediatric Dentistry (EAPD) and the MIH/HPSMs short charting form was used to score the index teeth. The results showed that the prevalence of MIH in Syrian children was 39.9%. Demarcated opacities were the most prevalent pattern of MIH defects on Permanent first molars (PFMs) and permanent incisors (PIs). *Spearman rank correlation* showed that the mean number of PIs and HPSMs with MIH increased when the number of affected PFMs was increased (*P* < 0.001). *Chi-square* test resulted that girls showed a higher number of severe PFMs than boys did with a statistically significant difference (x^2^ = 133.1, *P* < 0.05). Moreover, *Chi-square* test showed that the number of severe PFMs is higher than the number of severe PIs with a statistically significant difference (x^2^ = 54.9, *P* < 0.05). In addition, the mean dmft/DMFT index in children with MIH was found to be significantly higher than children without MIH (*P* < 0.05). The findings emphasize the need for early identification and management of MIH in children to prevent adverse effects on their oral health.

## Introduction

Molar-incisor hypomineralization (MIH) is a common developmental disorder that affects the permanent first molars and incisors of children. The condition is characterized by enamel hypomineralization, which results in discoloration, surface defects, and increased susceptibility to tooth decay^1,2^. MIH is a significant public health concern as it can lead to significant dental problems, including tooth loss and impaired function^3^.

The MIH is observed as asymmetrical, demarcated opacities on the permanent molars which vary in color from creamy- white to yellow–brown^4^.

Systemic in origin, MIH can affect one to four permanent first molars (PFMs), and in some cases, the permanent incisors are involved. Yellow/brown discoloration is seen as a more severe form of the condition compared to the white/creamy opacities, and the number of teeth affected as well as the severity of the defects can vary from individual to individual^4,5^.

MIH-like defects have also been detected on the second primary molars, second permanent molars, and tips of permanent canines^6^.

The hypomineralized enamel of teeth affected by MIH is more porous and has lower mechanical resistance, making it more vulnerable to dental caries as well as post-eruptive breakdown (PEB) when the affected tooth is under occlusal load. Atypical restorations (AR) may be observed in the affected teeth, with irregular margins and opacities around the restorations^7^.

Children with MIH may experience severe tooth sensitivity to temperature changes, making it difficult to maintain good oral hygiene and thus increasing the caries risk^8^.

The enamel affected by MIH can be a major challenge for dental practitioners, as it can be lost shortly after the tooth eruption, leading to dentin exposure and subsequent hypersensitivity. In some severe cases, the enamel requires complex treatment^9,10^.

Researchers reported a global prevalence of MIH ranging from 2.4 to 40.2%, with an estimated mean of 13.1 to 14.25%^11^. MIH is a disorder that has multiple causal factors, with systemic, genetic, and epigenetic factors interacting together to contribute to the development of the condition. This suggests that the etiology of MIH is multifactorial, with several factors working together. The evidence indicates that peri- and postnatal factors are more likely to be associated with MIH than prenatal factors, and they increase the likelihood of developing the condition^12^. However, more research is needed to provide further understanding about underlying etiological factors of MIH.

In Syria, the current crisis has resulted in a humanitarian catastrophe, with many children facing significant challenges to their health and well-being. The Syrian crisis has led to a breakdown of the economic system, which might have caused malnutrition in Syrian children affected directly by the crisis^13^.

The situation in Syria presents an opportunity to study the potential effects of the crisis on the prevalence, and severity of the MIH.

This cross-sectional study aimed to determine the prevalence, clinical pattern and severity of MIH among children in Syria, and to explore the association between MIH and dental caries.

## Materials and methods

### Study design and settings

This observational study was designed as an epidemiological cross-sectional study to investigate the prevalence of MIH in children aged 8–11 years old, which was taken place from the period of October and December 2021. Children were recruited from randomly selected public primary schools in Damascus city. All methods were carried out in accordance with all relevant guidelines and regulations and the ethical approval was obtained from the institutional Ethics committee of Damascus University (No. 2984). In addition, a written approval from the Ministry of Education was obtained. STORBE Statement guidelines were implemented to report this cross-sectional study. Informed consent was obtained from all participants guardians prior to the start of the data collection.

### Population sampling

To minimize selection bias, a multi-stage cluster sampling technique was used in this study. According to the Central Census Office in the Syrian Arab Republic, Damascus city is divided into 11 districts. Thus, two random public primary schools were selected from each district with a total number of 22 schools.

Multi-stage cluster sampling is a sampling method that involves dividing the population into clusters and selecting a sample of clusters using a random sampling technique. In this study, the population was primary school children in Damascus city. The first stage of the cluster sampling involved dividing Damascus city into 11 districts. Then, in the second stage, two public primary schools were randomly selected from each district, resulting in a total of 22 schools. In the third stage, all the students from grades (1, 2, 3) in the selected schools were invited to participate in the study.

Directors of each selected primary school were reached out in each district, and those that consent to participate were included in the research. Information about the study, including its purpose and methods, as well as a consent form and survey, were provided to the parents through an informational brochure.

### Inclusion and exclusion criteria

All participants in the study were selected based on the approval of their parents through a signed informed consent. Only boys and girls between the ages of 8 and 11, of a Syrian descent, and with at least one PFM erupted were eligible to participate. Uncooperative children and those who had orthodontic appliances were excluded from the study. Moreover, children with other enamel defects such as amelogenesis imperfecta or fluorosis were excluded.

### Examiner calibration

Before the start of this study, principle investigators (ZA, MA) were trained for the diagnosis of MIH using the short data form presented by Ghanim et al. in 2015^14^. The short data form was created specifically to evaluate the index teeth that are mentioned in the definitions of MIH and HPSMs, including the first permanent molars, permanent incisors, and second primary molars. This method assessed the severity of MIH and its impact on the affected tooth surface, other enamel defects, and presence of HPSMs in the same child.

The training included scoring photographs of both MIH and other enamel defects, which were repeated twice within a week. The inter- and intra-rater reliability were determined to be high, with Kappa statistics measuring 0.88 and 0.86 respectively. This ensured consistent and accurate examination of participants throughout the study.

### Study protocol and data collection

The clinical examination was performed individually, for each child using sterile mouth mirrors, WHO probes with a ball-shaped end (0.5 mm), gauze, and dental gloves on a chair with the head resting at 45 degrees to the horizontal.

A diagnosis of MIH was made for any case that had at least one affected first permanent molar. The examination took place in a room specifically prepared for the study in the participants’ schools.

This examination was carried out to all surfaces of the index teeth namely first permanent molars, permanent incisors, and second primary molars, after moisturizing them and removing plaque with gauze, under natural light and headlight. The headlight was a portable device with three led lamps offering sufficient lighting to perform the dental examinations. These index teeth were scored according to the MIH/HPSMs short charting form developed by Ghanim et al.^14^ in which teeth with clinical crown visible of 1/3 or more were scored.

In addition, the EAPD Classification system was used to evaluate the severity of MIH lesions in the study.

Mild lesions have been characterized by demarcated enamel opacities without breakdown. They only induced sensitivity to external stimuli and had only minor aesthetic concerns related to discoloration of the incisors. Severe lesions had demarcated enamel opacities with breakdown, caries, had significant aesthetic concerns that may have socio-psychological impacts, spontaneous and persistent hypersensitivity affecting functions.

### Statistical analysis

In this study, Descriptive data including means, frequencies in boys and girls, severity, percentages of affected surfaces and relative distributions of MIH were calculated. The DMFT index developed by the World Health Organization was also calculated after documenting the decayed, missing, and filled teeth.

The collected data were analysed using IBM SPSS software v. 23 (IBM corp., Armonk, USA). The *P* value of less than 0.05 was considered significant and the power of study were set at 90%. Independent *t* test was performed in order to compare between boys and girls in the frequencies of MIH. *Chi-square* test was used also to study the difference between boys and girls regarding the severity of MIH and to compare between the severity of affected molars and incisors. Moreover, the correlation between the number of index teeth (PFMs, PIs and HPSMs) was studied using *spearman’s rank correlation* with the level of significance of 0.001. Sample size was calculated using the following formula: $\frac{{z}^{2}p(1-p)}{{d}^{2}}deff$ which was recommended to determine sample size in descriptive cross-sectional studies^15^.

The standard normal variate at 5% type I error (1.96) was used to calculate the sample size. The proportion of the population was determined based on previous studies, with the higher global mean prevalence (14.2%) being chosen. The desired margin of error (d) was set to 3% and the design effect (deaf) was 2. The sample size calculation resulted in the need for 1040 participants, but 1138 children were actually recruited for the study.

## Results

During the study, 102 children were excluded from the analysis due to incomplete records (Consent form was not brought back or children missing the day of examination), which resulted in a final sample of 1138 children. The dropout rate was 8.96%, which is within an acceptable range for this type of study. The demographic characteristics of the excluded children were similar to those included in the analysis, indicating that the final sample was representative of the target population.

A total of 1138 children were recruited for this study, with an almost equal distribution of boys and girls. The mean age of the children was 8.9 years ± 2.1. The overall prevalence of MIH in the sample was 39.9% as 452 children out of the total examined sample (1138) suffered from MIH. The prevalence of MIH was higher in girls than in boys (41.9% vs 37.8%, respectively). However, Independent *t *test showed that there was no statistically significant difference between boys and girls regarding MIH suffering (t = 0.89*, P* > 0.05) (Table 1).

**Table 1:** Descriptive statistics of the sample.

	Boys	Girls
Number of children recruited	544	594
Number of children affected by MIH (%)	206 (37.8%)	246 (41.9%)
Age (Mean ± SD*)	9.2 ± 1.9	8.6 ± 2.2

*MIH* molar incisor hypomineralization.

*Standard deviation.

In regards to the distribution of MIH defects on the teeth, it was found that the majority of children (158 children, 35%) had two molars affected, (113 children, 25%) had one molar affected, (104 children, 23%) had three molars affected and (77 children, 17%) had four molars with MIH.

Moreover, 36% of the MIH-affected children had only affected PFMs (n = 163 children), 64% had affected permanent incisors (PIs) (n = 289 children) and 39% had hypomineralized primary second molars (HPSMs) (n = 176 children) simultaneous.

*Spearman rank correlation* showed that the mean number of PIs with MIH increased when the number of affected molars was increased (P < 0.001). Similarly, it was found that the mean number of HPSMs increased with increasing the number of affected permanent molars with MIH (P < 0.001). The findings are presented in Table 2.

**Table 2:** Spearman rank correlation between affected PFMs, PIs and HPSMs.

N. MIH-PFMs	Mean of MIH-PIs	Spearman rank correlation	*P*-value	Mean of HPSMs	Spearman rank correlation	*P*-value
1	1.73	0.762	< 0.001*	0.57	0.732	< 0.001*
2	2.09			1.01		
3	2.33			1.42		
4	2.89			1.98		

*N* number, *MIH* molar incisor hypomineralization, *PFMS* permanent first molars, *PIs* permanent incisors, *HPSMs* hypomineralized primary second molars.

*Statistically significant.

Based on the criteria of the severity of MIH according to the EAPD, mild MIH was diagnosed in 644 PFMs (61.3%) and severe MIH was diagnosed in 405 PFMs (38.6%). Furthermore, mild MIH was detected in 405 PIs (80.3%) and severe MIH was detected in 99 PIs (19.6%).

*Chi-square* test resulted that girls showed a higher number of severe PFMs than boys did with a statistically significant difference (x^2^ = 133.1, *P* < 0.05) (Table 3).

**Table 3:** Severity of MIH among boys and girl.

	Boys	Girls	x^2^	*P*-value
Mild PFMs	290	354	133.1	0.0001*
Severe PFMs	54	351		
Mild PIs	200	205	0.27	0.602
Severe PIs	46	53		

*MIH* molar incisor hypomineralization, *PFMS* permanent first molars, *PIs* permanent incisors.

*Statistically significant.

Moreover, *Chi-square* test showed that the number of severe PFMs is higher than the number of severe incisors with a statistically significant difference (x^2^ = 54.9, *P* < 0.05).

In addition, the extent of MIH defects on the index teeth was studied and it was found that the percentage of Grade 1 lesions (< 1/3 of the tooth surface) was higher than other grades in both permanent incisors and primary second molars. However, the percentage of Grade 2 lesions (at least 1/3 but < 2/3 of the tooth surface) was the highest (45%) in the MIH permanent first molars (Table 4).

**Table 4:** Proportion of tooth surface affected by MIH lesion in index teeth.

	PFMs	PIs	HPSMs
Grade 1*	29%	60%	44%
Grade 2**	45%	35%	39%
Grade 3***	26%	5%	17%

*MIH* molar incisor hypomineralization, *PFMS* permanent first molars, *PIs* permanent incisors, *HPSMs* hypomineralized primary second molars.

*Grade I < 1/3 of the tooth surface.

**Grade II atleast 1/3 but < 2/3 of the tooth surface.

*** Grade III atleast 2/3 of tooth surface.

Regarding the EAPD diagnostic criteria, demarcated opacities were the most MIH lesions affecting PFMs, PIs and HPSMs. Atypical caries were detected more in HPSMs, followed by PFMs and PIs (31.6%, 20% and 2.3%) respectively (Table 5).

**Table 5:** Distribution of EAPD diagnostic criteria in index teeth.

EAPD diagnostic criteria	PFMs		PIs		HPSMs	
	N	%	N	%	N	%
Demarcated opacities	576	54.9	403	80	345	51.4
Post-eruptive enamel breakdown	137	13	70	13.8	64	9.5
Atypical restoration	104	9.9	19	3.7	32	4.7
Atypical caries	210	20	12	2.3	212	31.6
Atypical extraction	22	2.09	0	0	17	2.5
Total	1049	100	504	100	670	100

*PFMS* permanent first molars, *PIs* permanent incisors, *HPSMs* hypomineralized primary second molars.

The mean dmft index for primary teeth in children with MIH was found to be 4.66 ± 2.2, which is significantly higher than the mean dmft index of 2.90 ± 1.1 in children without MIH (t = 2.21, *P* < 0.05). In addition, the mean DMFT index for permanent teeth in children with MIH was found to be 3.05 ± 1.8, which is significantly higher than the mean DMFT index of 1.44 ± 0.5 in children without MIH (t = 2.95, *P* < 0.05). Table 6 demonstrates the descriptive data of dmft index and DMFT index for children with and without MIH.

**Table 6:** Mean dmft and DMFT index in children with and without MIH.

	MIH group	Non-MIH group
d	2.48 ± 1.9	1.06 ± 0.8
m	0.56 ± 0.2	0.88 ± 0.3
f	1.62 ± 0.8	0.96 ± 0.4
dmft	4.66 ± 2.2	2.90 ± 1.1
D	1.98 ± 0.6	0.77 ± 0.4
M	0.31 ± 0.1	0.0 ± 0.0
F	0.76 ± 0.2	0.67 ± 0.3
DMFT	3.05 ± 1.8	1.44 ± 0.5

## Discussion

The present study aimed to investigate the prevalence of MIH in Syrian children and its clinical characteristics. A cross-sectional study design was employed and a sample of 1138 children was recruited from different regions in Damascus, Syria. The study found a high prevalence of MIH in Syrian children in Damascus city, with a rate of 39.9%.

In this study, the European Academy of Paediatric Dentistry (EAPD) criteria were utilized to diagnose MIH and index teeth were scored using the MIH/HPSMs short charting form by Ghanim et al.^14^. This form was recommended by the recent EAPD policy document about the best clinical practice guidance for dealing with children presenting with MIH^16^.

The EAPD criteria are based on clinical observations and take into account the presence of hypomineralized white or brown opacities on the incisal or occlusal surfaces in the enamel of permanent molars and/or incisors, which are the main clinical features of MIH^16^.

The criteria also exclude other possible causes of developmental defects, such as cavitation of the affected tooth surfaces, history of systemic disease, previous treatment, and excessive fluoride exposure.

In addition, the EAPD criteria provide a grading system for the severity of MIH, which can be used to classify affected teeth as mild or severe. This allows for a more accurate and consistent assessment of the condition and better comparison between studies^16,17^. Using the EAPD criteria in this study allowed us to perform consistent and accurate assessment and to provide number about the prevalence of MIH in the sample of children aged 8 to 11 years.

This age range is associated with the eruption of permanent first molars and incisors, which are most affected teeth by MIH, and thus provided us with a more accurate estimate of the prevalence of MIH^16,17^. In addition, children in this age group are more cooperative and amenable to a dental examination, which facilitated the data collection process. It’s worth mentioning that this age group was selected as it represents the age group that is most susceptible to suffering from MIH, as the majority of MIH cases are seen in permanent teeth^18^.

In this study, the prevalence of MIH in Syrian children aged between 8 and 11 years from Damascus city was 39.9%, with no significant gender predilection.

It is evident that the prevalence of MIH in Syrian children from Damascus city is higher than in other countries in the region. A study conducted in Jordan reported a prevalence of 13.2%^19^, another study in Saudi Arabia reported a prevalence of 15.2%^20^. While a study in Lebanon reported a prevalence of 26.7%^21^.

Comparing the results of this study to studies conducted in underdeveloped countries with similar socio-economic conditions to Syria can provide valuable insights into the factors that may contribute to the high prevalence of MIH in Syrian children in Damascus city.

A study conducted in Nepal, for example, reported a prevalence of MIH of around 12.9%^22^, which is significantly lower than the rate found in this study. Similarly, a study conducted in Sudan reported a prevalence of MIH of around 20.1%^23^.

These findings suggest that the prevalence of MIH in Syrian children may be unique to the country, possibly due to the effects of the ongoing Syrian crisis on the population’s health. The crisis may have resulted in a lack of access to healthcare services, as well as poor living conditions that may have affected the oral health of children in Syria^24,25^.

The Syrian crisis could potentially affect the prevalence of MIH in Syrian children. The crisis has led to significant disruptions in the healthcare system and access to medical care in Syria, which could potentially affect the incidence and diagnosis of MIH. Additionally, the crisis has led to a displacement of a large number of Syrian people, which could lead to changes in children’s and their mothers’ living conditions, diet, and access to clean water, which are all potential risk factors for MIH^26–28^. Moreover, factors such as stress, malnutrition, and exposure to environmental toxins, which are all associated with the Syrian crisis, could also affect the development of children’s teeth and lead to an increased incidence of MIH^29^.

It is important to consider the impact of the Syrian crisis on oral health and potential changes in the prevalence of MIH in Syrian children when undertaking research on the disorder in this population.

The results of this study showed that there was no significant difference between Syrian male and female children in the prevalence of MIH which was consistent with other studies that showed that MIH can effect both genders without predilection^19–21,23^.

This finding suggests that MIH is not gender-specific, and both boys and girls are equally likely to develop this condition^30^. This information is valuable in understanding the impact of MIH on paediatric dental health and in providing equitable care for all children. The lack of a gender-based difference in MIH prevalence also highlights the importance of considering other risk factors, such as environmental exposure, nutritional deficiencies, and medical conditions, when assessing the cause and development of MIH.

In this study, it was observed that 36% of children diagnosed with MIH had only their permanent molars affected, while the rest had both molars and incisors involved. This finding highlights the variable presentation of MIH in children and its potential impact on both anterior and posterior teeth. This result also indicates the need for a comprehensive examination of all teeth in children with suspected MIH, rather than just focusing on a single affected tooth or area^31^. This information can be valuable for dental practitioners to better understand the pattern of MIH in children and provide comprehensive and effective care for these children.

The findings of this study suggested a strong correlation between the number of molars affected MIH and the number of affected PIs and HPSMs which was consistent with the finding of other studies^20,23,32^. This outcome highlights the systemic nature of MIH and its potential to have a significant impact on a child’s oral health. The higher the number of molars affected, the greater the likelihood that other teeth in the oral cavity will also be affected by MIH.

This information is crucial for dental professionals as it highlights the need for early detection and intervention to prevent the negative effects of MIH and protect the oral health of children. Further studies are still required to further understand the underlying mechanisms behind this correlation in order to develop effective preventive and management strategies for MIH.

Regarding the severity of MIH lesions, the results of this study showed that the severity of MIH was higher in molars compared to incisors. This finding is consistent with previous studies, which indicated that MIH is more frequent and severe in molars than incisors^32,33^.

In fact, molars are subject to more occlusal stress during chewing and grinding, which could increase the risk of hypomineralization. Additionally, molars have longer developmental periods and more complex morphologies compared to incisors, making them more susceptible to MIH^18^. The higher severity of MIH in molars is a cause for concern as molars play a crucial role in biting and chewing, and their loss or damage can have a significant impact on oral function and quality of life. Thus, it is important to develop strategies to prevent or manage MIH in molars.

In addition, the results of the current study suggested that there is a significant difference in the severity of MIH between female and male children. Specifically, the findings showed that the severity of MIH is higher in girls compared to boys. This difference could be attributed to the faster rate of tooth eruption in girls^20,34^. Previous research has demonstrated that the earlier eruption of teeth can increase the susceptibility to hypomineralization due to the longer exposure to risk factors^30^.

As a result, it is important for dental professionals to be aware of the higher MIH severity in girls in the Syrian population and to consider this factor when developing preventive and therapeutic strategies. Further studies are still essential to explore the underlying mechanisms that contribute to the gender differences in MIH severity.

Moreover, this study indicated that the most common clinical pattern of MIH in Syrian children was demarcated opacities. This pattern is characterized by a white or yellowish discoloration, chalky appearance, and/or rough surface on the affected teeth. The prevalence of this pattern is consistent with previous studies conducted on MIH in children from different countries^19,21,23^, indicating that demarcated opacities are a common feature of MIH lesions in paediatric populations globally. The observed pattern highlights the need for early detection and management of MIH in Syrian children, as the severity and extent of the lesions can significantly impact the oral health and quality of life of affected individuals^11^.

One of the key findings of this study was that children with MIH tended to have a higher DMFT (Decayed, Missing, and Filled Teeth) index compared to those without MIH. This highlights the negative impact that MIH can have on oral health and raises important questions about the underlying causes of this disparity. The presence of mild enamel lesions in MIH-affected molars may make children more susceptible to dental decay, as the softer and more porous enamel may be more prone to caries formation^8,35^. Additionally, children with MIH may experience more discomfort or pain associated with their teeth, which could impact their oral hygiene habits and increase the likelihood of developing dental problems^36^. Further work should explore these potential explanations and develop effective strategies for preventing MIH in children.

It is important to note that this study has some limitations, as it does not allow causal inferences to be drawn and did not assess the quality of oral health care, socio-economic status, or other environmental factors that could be associated with MIH. Finally, it should be noted that this study was conducted in one city only (Damascus). While the sample size was large and diverse, the results may not necessarily be representative of other populations or regions. Future research should aim to replicate these findings in other locations to establish the generalizability of the results.

It is speculated that the high prevalence of MIH in Syrian children in Damascus city could be a result of the Syrian crisis. The causation ought to be further analyzed and the timing of mineralizing FPM has to be considered. Therefore, future work should consider exploration of the potential factors that contribute to high prevalence of MIH in Syrian children during the Syrian crisis.

## Conclusion

The prevalence of MIH in Syrian children aged between 8 and 11 years from Damascus city was relatively high, with no significant gender predilection. Severe cases of MIH were more common in molars than incisors and more frequent in girls than boys. The most prevalent pattern of MIH defects detected in PFMs and PIs in this study was the demarcated opacities. There was a significant association between MIH and dental caries**.** Future work should further investigate the etiological factors behind high prevalence of MIH cases among Syrian children during the crisis to provide further understanding in order take preventive and management measures.

## Data Availability

The data provided for the results presented in this study is available through the corresponding author upon request.

## Abbreviations

MIH: Molar incisor hypomineralization
PFMs: Permanent first molars
PIs: Permanent incisors
HPSMs: Hypomineralized primary second molars
PEB: Post-eruptive enamel breakdown
AR: Atypical restoration
SPSS: Statistical package for social sciences
EAPD: European academy of paediatric dentistry
